# Hahn-Steinthal fracture: a case report

**DOI:** 10.1186/1757-1626-1-239

**Published:** 2008-10-15

**Authors:** Shishir P Nawghare, Rudraprasad Baidyaray, JGV Neyt

**Affiliations:** 1 Department of Orthopaedics and Trauma, Chase Farm Hospital, Enfield, UK

## Abstract

Isolated fracture of the capitellum is rare. We present clinical and radiological data on a single case of a fracture of capitellum. We came across a 31 year old woman who sustained an isolated Hahn Steinthal type of fracture. It was treated operatively by open reduction and internal fixation using mini fragment screws. The elbow was immobilized for 4 weeks. The patient regained full range of movement at 12 weeks post operatively. We reiterate that anatomical reduction and fixation is the right way to treat this injury.

## Introduction

Fracture of the capitellum is uncommon. Since fracture of the capitellum is rare, most of the information in the available literature is based on only a few cases. They account for 6% of distal humerus fractures [1-3]. We present a case of a 31 year old woman who presented with a Type I (Hahn-Steinthal) fracture of the capitellum. The fracture was treated by open reduction and internal fixation. The result of this form of management was found to be satisfactory.

## Case presentation

**Figure 1:**
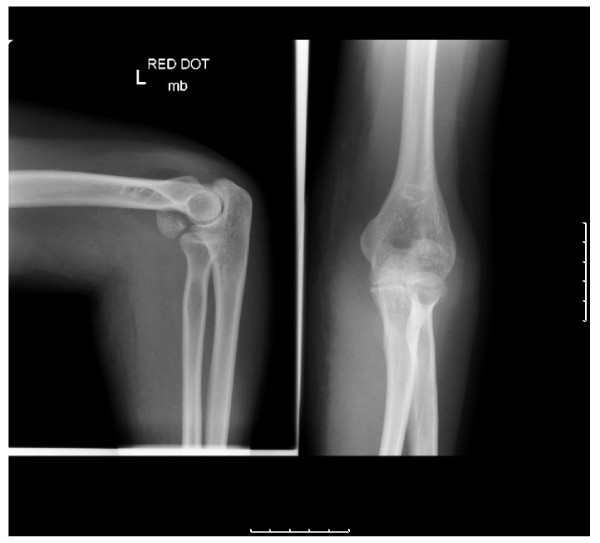
Radiograph of capitellar fracture.

**Figure 2:**
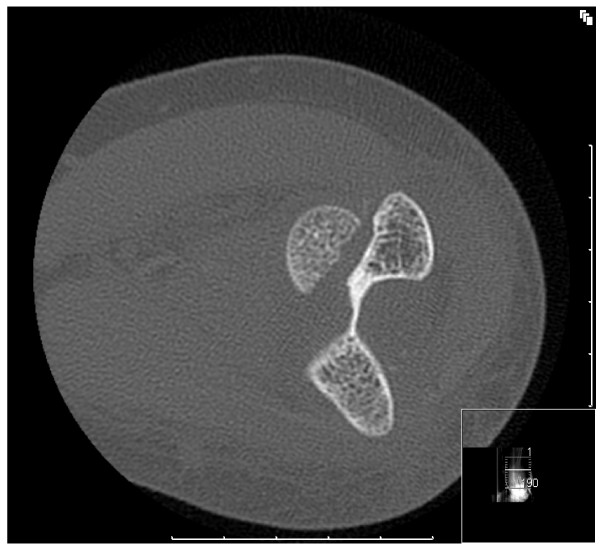
CT scan of capitellar fracture.

**Figure 3:**
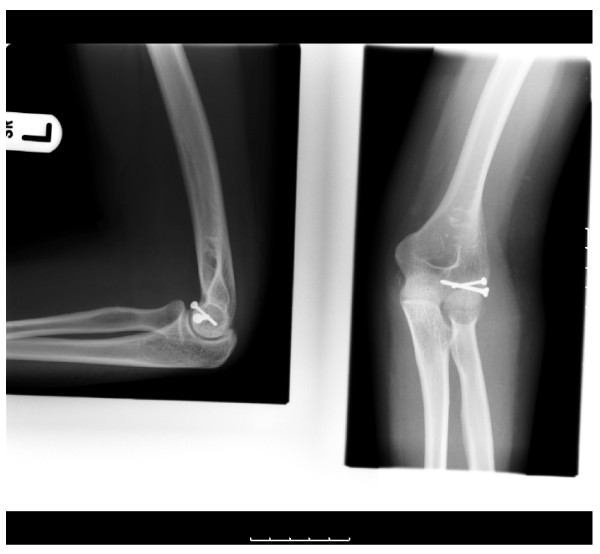
Radiograph after fixation.

A 31 year old right handed lady of Afro-Carribean origin presented to the accident & emergency department with a history of fall on her left elbow. There was pain and swelling around the elbow. The movements at the elbow were painful and restricted. There was no neurovascular deficit. The radiographs(Fig 1) revealed a fracture of the capitellum which was reconfirmed as an isolated Type I (Hahn-Steinthal) fracture by a CT scan(Fig 2). A decision for open reduction and internal fixation was taken. Using the postero-lateral approach as described by Kocher, the fracture was fixed using two 2-0 mm minifragment screws. The elbow was immobilized in a plaster for 4 weeks. This was followed by a progressive elbow mobilization programme guided by the physiotherapist. She was followed up at 4 weeks, 6 weeks, 3 months, 6 months and 12 months. She attained full range of movement at 3 months(Fig 3) with no further complications later.

## Discussion

The first description of capitellar fracture was put forth by Hahn [4] and Steinthal [5] in the 19th century. This fracture is more common in individuals older than 12 years and very rare in children. A fall on the outstretched hand or directly on the elbow produces a shear force fracturing the capitellum in the coronal plane. As the center of rotation of the capitellum is 12–15 mm anterior to the humeral shaft, it is vulnerable to the shear forces.[6] These fractures can be classified according to the McKee modification of the Bryan and Morrey classification. [2,7] Type I (Hahn-Steinthal) is a coronal shear fracture with a large osseous capitellar fragment [4,5] Type II involves a shell of the articular cartilage with a thin layer of bone and are known by the eponym Kocher-Lorenz [8,9]. Type III fractures include all comminuted fractures of the capitellum. [2] McKee et al[7] added a fourth pattern, noting that in some cases the Hahn-Steinthal[4,5] fracture extends medially in the coronal plane to include the lateral half of the trochlea. There is no universal agreement on the treatment of this fracture. Closed reduction of type I has been advocated [10]. It can be treated surgically by open reduction and internal fixation using minifragment standard screw set, Kirschner wires (K-wires), small/minifragment Herbert screws, absorbable pins, compression screws, staples and bone pegs. The treatment of type II & III involves excision of the of the fragments as fixation is not feasible. Isolated fracture of capitellum is indeed a rare injury. The treatment of the fracture is still controversial. There is no randomized controlled trial available to direct the correct line of management. Working along the good principles of fracture management, we reduced the fracture after exposing it and fixed it with mini fragment screws. We conclude that reconfiguring the anatomical exactness is perhaps the best form of treatment for the Hahn Steinthal fracture. To this effect, fixing the fracture with mini fragment screws after open reduction is definitely the way forward. Although we used the mini fragment screws for fixation, we agree that any form of fixation which helps reconstruct the anatomy perfectly is acceptable.

## Competing interests

The authors declare that they have no competing interests.

## Authors' contributions

All the authors have made substantial contributions to conception and design, or acquisition of data, or analysis and interpretation of data, have been involved in drafting the manuscript or revising it critically for important intellectual content; and have given final approval of the version to be published.

## Consent

Written informed consent was obtained from the patient for publication of this case report and accompanying images. A copy of the written consent is available for review by the Editor-in-Chief of this journal.

## References

[B1] Poynton AR, Kelly IP, O'Rourke (1998). Fractures of the capitellum: a comparison of two fixation methods. Injury.

[B2] Bryan RS, Morrey BF, Morrey BF (1985). Fractures of the distal humerus. The Elbow and its Disorders.

[B3] Marion J, Faysse R (1962). Fractures du capitellum. Rev Chir Orthop.

[B4] Hahn NF (1853). Fall von einer besonderen Varietat der Frakturen des Ellenbogens. Zeitschrift fur Wundarzte und Geburtshelfer.

[B5] Steinthal D (1898). Die isolierte Fraktur der Eminentia Capitata im Ellenbogengelenk. Zentralbl Chirurgie.

[B6] Ertl JP (2007). Capitellar Fracture. Emedicine.

[B7] McKee MD, Jupiter JB, Bamberger HB (1996). Coronal shear fractures of the distal end of the humerus. J Bone Joint Surg [Am].

[B8] Kocher T (1896). Beitrage zur kenntniss einger praktisch wishctiger fraktur formen. Mitheil a Klin u Med Inst & Schweiz Basal, reihe.

[B9] Lorenz H (1905). Zur kenntnis der fractural capitulum humeri (Eminentiae Capitatae). Dtsche Ztrschr f Chir.

[B10] Ochner RS, Bloom H, Palumbo RC, Coyle MP (1996). Closed reduction of coronal fractures of the capitellum. J Trauma.

